# Total parathyroidectomy plus multi-point subcutaneous transplantation in the forearm may be a reliable surgical approach for patients with end-stage renal disease: A case report

**DOI:** 10.1097/MD.0000000000017649

**Published:** 2019-11-22

**Authors:** Nan Lin, Yong Chao Fang, Jun Chuan Song, Yu Wang

**Affiliations:** a Department of General Surgery, Dongfang Hospital, Xiamen University.; b Department of General Surgery, Fuzhou General Hospital, Fuzhou, Fujian, China.

**Keywords:** multi-point subcutaneous transplantation, secondary hyperparathyroidism, surgical approach

## Abstract

**Rationale:**

We studied the feasibility of total arathyroidectomy(tPTX)+multi-point transplantation in the forearm for treatment of secondary hyperparathyroidism. Considering the controversial nature of the appropriate timing for and location of this type of surgery, relevant research is relatively rare. Our experience may be a relatively successful one.

**Patient concerns:**

Our patient was a 28-year-old woman with end-stage renal disease (ESRD), who was on dialysis for 7 years, and a 2-year history of progressively aggravated bone pain. She also had hypercalcemia and hyperphosphatemia.

**Diagnoses:**

Given the patient's history of long-term dialysis, bone pain, high levels of intact parathyroid hormone(i-PTH) and hypercalcemia, we performed ultrasonography which showed solid nodules in the bilateral parathyroid glands. She was accordingly diagnosed with SHPT.

**Interventions:**

The patient underwent tPTX+multi-point subcutaneous transplantation in the forearm.

**Outcomes:**

Her i-PTH level dropped to < 300 pg/mL, and the symptoms of bone pain markedly reduced after surgery.

**Lessons:**

Total parathyroidectomy+multi-point subcutaneous transplantation in the forearm may be a reliable surgical approach for patients with ESRD.

## 1 Introduction

Secondary hyperparathyroidism (SHPT)is one of the major complications in patients with endst-age renal disease (ESRD) maintained on dialysis.^[1]^ Medication with oral calcitriol, vitamin D analogues, and calcimimetics may lead to an overall decrease in parathyroidectomies (PTXs); however, PTX is still a good choice. Komaba et al. showed in his nationwide cohort of maintenance hemodialysis patients that those who underwent PTX had remarkable survival advantage compared with the matched control with severe SHPT that did not undergo PTX.^[2]^ At present, there are 3 options for the surgical treatment of severe SHPT: subtotal PTX, total PTX with autotransplantation, and total PTX without autotransplantation. A prospective randomized trial^[8]^ that compared total PTX+autotransplantation with subtotal PTX showed better results for the former in terms of postoperative levels of calcium, phosphate, alkaline phosphatase, as well as an improvement in clinical symptoms and osteopathy. Reoperations after total PTX+autotransplantation were less common, and those performed had a lower complication rate.^[3]^

## 2 Case report

Written informed consent was obtained from the patient for the publication of this manuscript and accompanying images.

**Figure 1:**
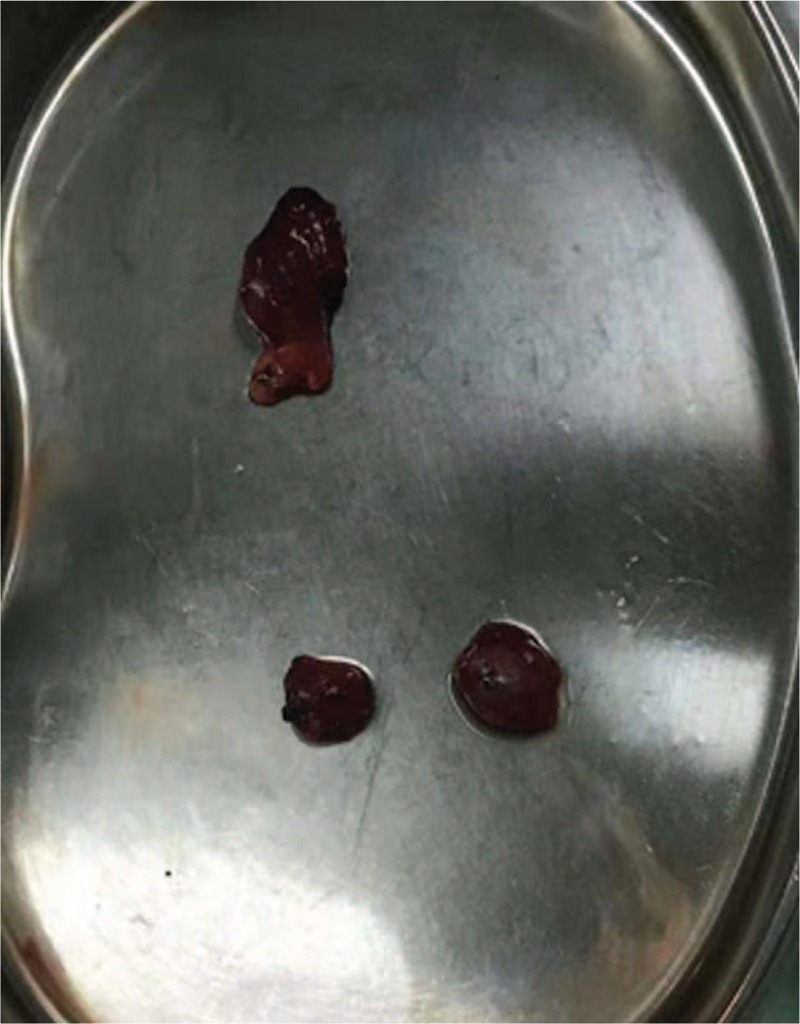
Three enlarged parathyroid glands that were removed during surgery.

**Figure 2:**
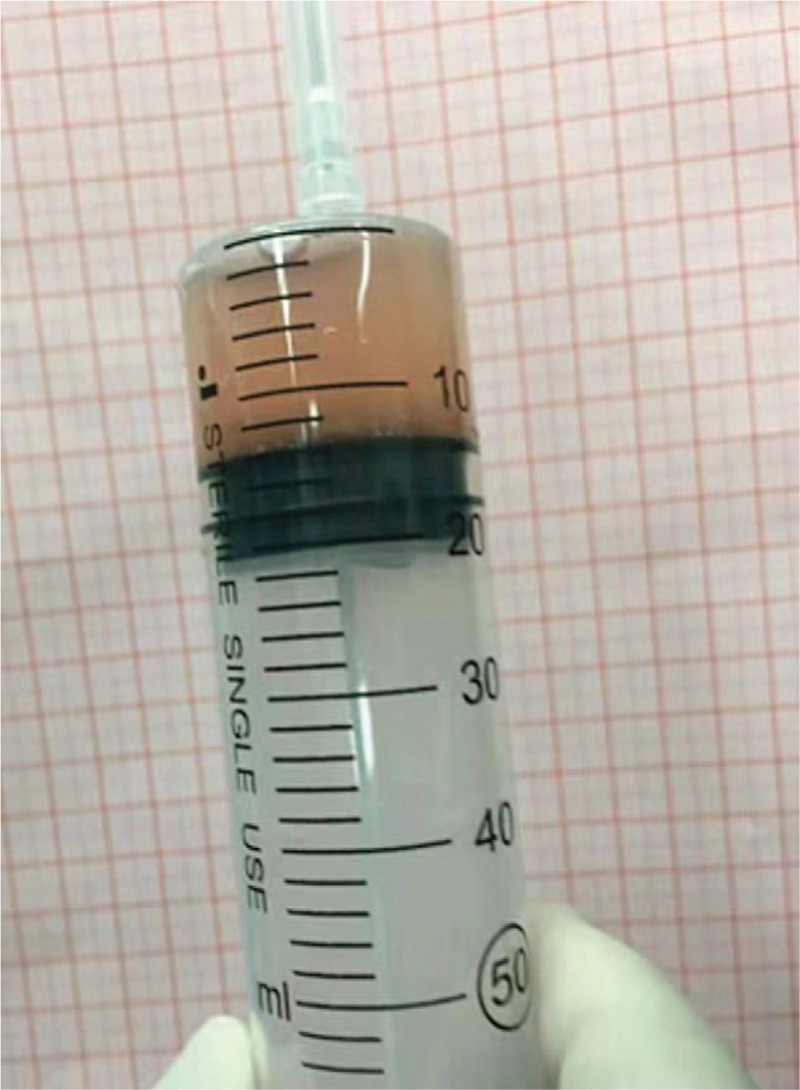
The parathyroid serum was made using the smallest parathyroid gland.

**Figure 3:**
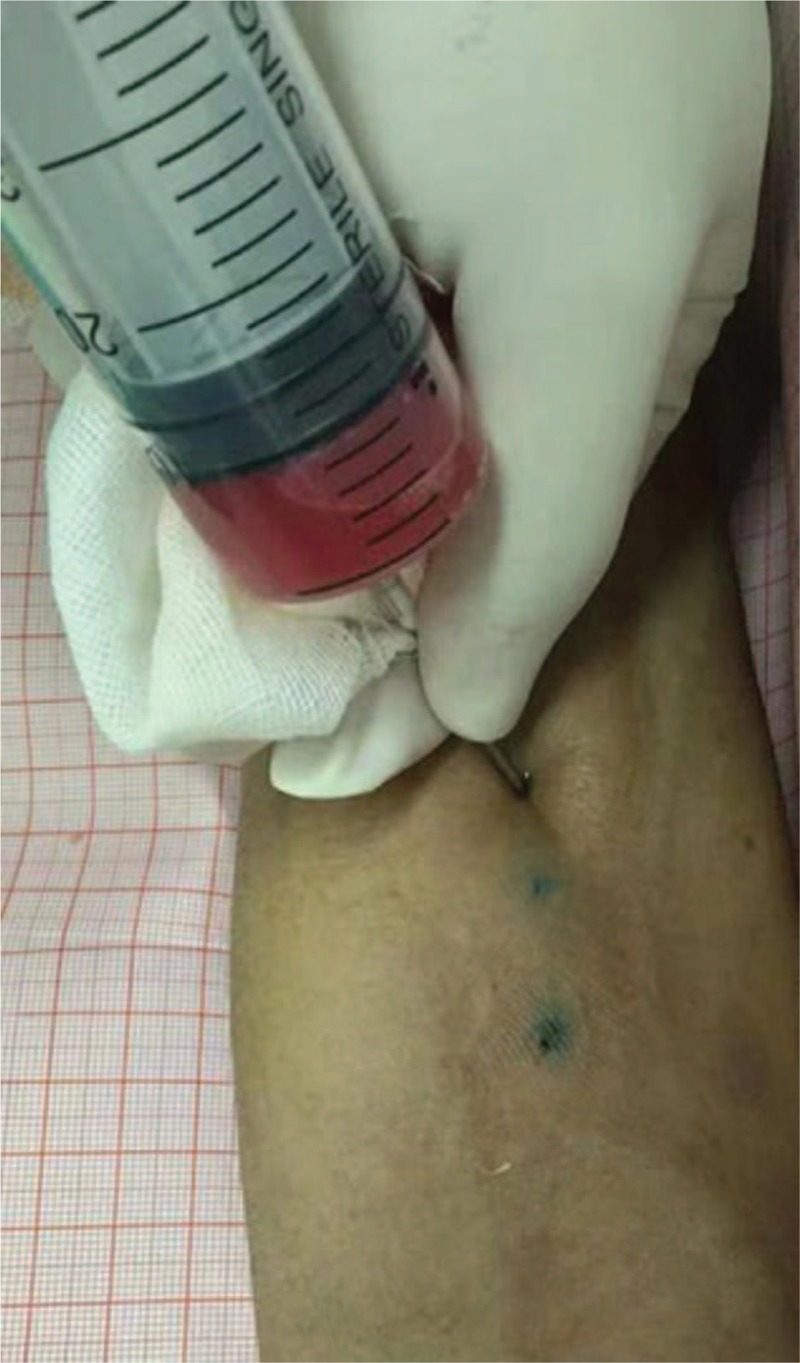
The parathyroid serum was injected into the subcutaneous area of the right forearm at 3 points.

A 28-year-old woman with ESRD on dialysis for 7 years and with a 2-year history of progressively aggravating of bone pain presented to us. Preoperatively, her intact parathyroid hormone (i-PTH) level was >2500 pg/mL, ionized calcium level was 3.05 mmol/L and serum phosphate level was 1.75 mmol/L. Ultrasound detected no abnormalities with the thyroid or lymph nodes, but three hypoechoic areas were seen in the parathyroid area. The 99mTc-MIBI three-phase imaging confirmed the presence of bilateral, irregular masses under the thyroid lobes on the 15- and 120-minute images. A diagnosis of SHPT was established. The operation time was about 100 minutes. After induction of general anesthesia, we made an arc-shaped incision 3-cm above the sternal notch, freed the flaps layer by layer, cut the anterior cervical anterior muscle group, and exposed the thyroid gland. We could also see the rear abnormal parathyroid gland. Then, we separated the parathyroid gland from the normal thyroid tissue along the envelope and completely removed the enlarged parathyroid gland. Three enlarged parathyroid glands were found during the surgery; all were removed, (Fig. 1), and the smallest parathyroid gland was cut and used to make a parathyroid serum (Fig. 2) that was injected into the subcutaneous area of the right forearm at 3 points (Fig. 3). The thyroid gland appeared normal. After the transplantion, the operator applied pressure to the injection site for 5 minutes.

Postoperatively, her symptoms of bone pain had significantly alleviated, and her i-PTH levels decreased to 116 pg/mL, 51 pg/mL and 27.4 pg/mL at 30 and 60 minutes and 24 hours after surgery, respectively. Her serum phosphate level gradually dropped to the normal range, but symptomatic hypocalcaemia increased postoperatively, which required intravenous supplements, although the patient appeared well. Following surgery, her bone pain and other symptoms significantly improved; thus, she was discharged from the hospital 7 days after the operation. Surprisingly, the patient developed obvious bone pain on the 10th day after surgery and could not walk; moreover, her i-PTH level increased to 935 pg/mL. We closely monitored the patient's hormone levels and clinical symptoms, and initiated symptomatic analgesic treatment and ensured that she was comfortable. On the 17th day after surgery, her i-PTH level dropped below 300 pg/mL, and the symptoms of bone pain markedly reduced. At the 3-month follow-up, we did not find any significant complications.

## 3 Discussion

While PTX is a classic surgical procedure, it can cause hypocalcemia. The only remedial option is long-term oral calcium or intravenous calcium supplementation. Subtotal PTX often does not effectively reduce levels of i-PTH and ionized calcium. As SHPT recurrence may be induced by autotransplanted parathyroid glands, tPTX+ autotransplantation, which involves transplantation of flakes of or powdered parathyroid gland into the forearm muscles or the sternocleidomastoid muscle, resulting in the patient receiving a second operation. But tPTX+multi-point subcutaneous transplantation in the forearm can avoid these problems. To our best knowledge, there are no other similar reports yet. The patient fully understood and accepted the operation and provided written informed consent for the same. We acknowledge that this procedure has some limitations. There are currently not enough cases to confirm the reliability of our surgery. Surgical treatment for SHPT may be needed for more in-depth research to provide greater benefits to patients with ESRD.

Treatment success was defined as i-PTH *<*300 pg/mL.^[4]^ Therefore, we believe that the operation was very successful. Unexpectedly, on the 10th day after surgery, the patient developed obvious bone pain and could not walk, the i-PTH level increased to 935 pg/mL. We suspected that it may be the result of self-regulation such as the balance of calcium and phosphorus or the interaction between osteoblasts and osteoclasts. We are also aware that it may take several days to reach equilibrium despite successful symptomatic treatment. About a week later, her i-PTH level dropped to <300 pg/mL, and the symptoms of bone pain markedly improved. A month later, she only experienced mild residual bone pain upon walking. Overall the patient's symptoms have been greatly alleviated, despite a transient increase in the i-PTH level and increased bone pain after surgery. The surgical procedure of tPTX+ autotransplantation, which involves resection of all parathyroid glands, with the smallest and most “normal” one cut into flakes or powdered, followed by transplantation 60 to 100 mg into the forearm muscles (one side of the upper limb without arteriovenous fistula) or the sternocleidomastoid muscle, and possibly even subcutaneous tissue.^[5]^ This method avoids postoperative hypocalcemia, and can effectively alleviate the symptoms, but also has limitations, as recurrence of SHPT may be induced by the autotransplanted parathyroid glands.^[6,7]^ While our surgical approach is relatively novel, we cut one of the removed parathyroid glands and transplant them separately to the forearm. Transplantation into the subcutaneous area is beneficial for B-ultrasound-based detection during later follow-up. For recurrent or persistent severe SHPT, reoperation is an invasive procedure with increased incidence of complications,^[8]^ and might not even result in successful treatment for those patients.^[9]^ If sHPT recurs, we can consider ablation of 1 or 2 of the 3 transplant points on the right forearm. The ablation does not require high operational skills.

In conclusion, we reasoned that tPTX+multi-point subcutaneous transplantation in the forearm may be a reliable surgical approach to treat SHPT. Patients with ESRD diagnosed with SHPT and having hypercalcemia, hyperphosphatemia, and high levels of i-PTH are suitable candidates for this procedure. A prospective randomized trial showed that percutaneous ethanol injection therapy (PEIT) is useful for treating recurrent and persistent hyperparathyroidism after PTX.^[4]^ Because of multi-point transplantation, surgical removal of transplanted parathyroid glands is also feasible for this patient, if the condition recurs.

## Author contributions

**Writing – original draft:** Nan Lin, Yong Chao Fang, Jun Chuan Song, Yu Wang.
